# Preoperative NT-proBNP and LVEF for the prediction of acute kidney injury after noncardiac surgery: a single-centre retrospective study

**DOI:** 10.1186/s12871-022-01727-0

**Published:** 2022-06-24

**Authors:** Jiaqi Wang, Yehong Dong, Bingcheng Zhao, Kexuan Liu

**Affiliations:** Department of Anaesthesiology, Nanfang Hospital, Southern Medical University, 1838 Guangzhou Avenue North, Guangzhou, 510515 China

**Keywords:** Acute kidney injury, Anaesthesia, Noncardiac surgery, N-terminal pro-B-type natriuretic peptide, Left ventricular ejection fraction, Risk assessment

## Abstract

**Background:**

Acute kidney injury (AKI) is one of the most common postoperative complications in noncardiac surgical patients, has an important impact on prognosis and is difficult to predict. Whether preoperative N-terminal pro-brain natriuretic peptide (NT-proBNP) concentrations and left ventricular ejection fraction (LVEF) levels can predict postoperative AKI in noncardiac surgical patients is unclear.

**Methods:**

We included 3,314 patients who underwent noncardiac surgery and had measurements of preoperative NT-proBNP concentrations and LVEF levels at a tertiary academic hospital in China between 2008 and 2018. Multiple logistic regression analysis was used to construct a postoperative AKI risk prediction model for this cohort. Then, NT-proBNP concentrations and LVEF levels were included in the abovementioned model as independent variables, and the predictive ability of these two models was compared.

**Results:**

Postoperative AKI occurred in 223 (6.72%) patients within 1 week after surgery. Preoperative NT-proBNP concentrations and LVEF levels were independent predictors of AKI after adjustment for clinical variables. The area under the receiver operating characteristic curve (AUROC) of the AKI risk predictive model established with clinical baseline variables was 0.767 (95% CI: 0.732, 0.802). When NT-proBNP concentrations and LVEF levels were added to the base model, the AUROC was 0.811 (95% CI: 0.779, 0.843). The addition of NT-proBNP concentrations and LVEF levels improved reclassification by 22.9% (95% CI 10.5–34.4%) for patients who developed postoperative AKI and by 36.3% (95% CI 29.5–43.9%) for those who did not, resulting in a significant overall improvement in net reclassification (NRI: 0.591, 95% CI 0.437–0.752, *P* < 0.000). The integral discrimination improvement was 0.100 (95% CI: 0.075, 0.125, *P* < 0.000).The final postoperative AKI prediction model was constructed, and had a good discriminative ability and fitted to the dataset.

**Conclusions:**

Preoperative NT-proBNP concentrations and LVEF levels were independently associated with the risk of AKI after noncardiac surgery, and they could improve the predictive ability of logistic regression models based on conventional clinical risk factors.

**Trial registration:**

The protocol was preregistered in the Chinese Clinical Trial Registry (ChiCTR1900024056).

## Background

Acute kidney injury (AKI) is a common complication during the early postoperative period after noncardiac surgery. Some scholars have reported that the prevalence of AKI after thoracic surgery is approximately 12%, while the pooled incidence of AKI after abdominal surgery is 13.4% [1, 2]. Increased postoperative serum creatinine, even in those who did not develop renal disease, was associated with adverse outcomes, including increased morbidity, length of hospitalization, health care costs and short- and long-term mortality [3–6]. In such settings, the development of risk prediction models for AKI that could be used to risk stratify patients to develop avoidance, preventative or early treatment approaches was listed as a research recommendation in the Kidney Disease: Improving Global Outcomes (KDIGO) clinical practice guidelines for AKI. Currently, a substantial number of risk prediction models have been reported to predict AKI after cardiac surgery, and many of them have been externally validated in more than one independent cohort [7–15]. However, the prediction of AKI after major noncardiac surgeries has received much less attention, although the incidence of AKI ranges from 7 to 30% [16]. Only a few prediction models for AKI after noncardiac surgery have been explored in patients who underwent a single type of specialized surgery, which represents only a subset of noncardiac surgeries. Therefore, the value of these models is limited. Moreover, a recent study suggested the predictive ability of NT-proBNP concentrations for AKI in the perioperative period in patients who underwent living donor liver transplantation [17]. Another study found that patients with decreased left ventricular systolic function are at increased risk for postoperative infections and kidney injury, demonstrating a relationship between cardiac dysfunction and noncardiac complications after surgery [18]. Whether NT-proBNP concentrations and LVEF levels could provide additional predictive information beyond conventional AKI risk factors in a broader and heterogeneous group of noncardiac surgical patients is still unknown [19]. Therefore, considering the complex interactions between renal and cardiac dysfunction and that NT-proBNP concentrations and LVEF levels are good markers for assessing haemodynamic stress and cardiac dysfunction, we added preoperative NT-proBNP concentrations and LVEF levels to the preoperative risk prediction model for AKI following noncardiac surgery constructed using by the conventional AKI risk factors and determined whether they could improve the prediction ability.

## Methods

The clinical data of all patients were obtained from a tertiary hospital in Guangzhou, China. This study complied with the Declaration of Helsinki and was approved by the Ethics Committee of Southern Medical University Nanfang Hospital without the need for informed consent from the participants (NFEC-2019–081). Additionally, as this was a retrospective study, all identifying information of the patients was removed before analysis [19].

### Patients

The study was a secondary analysis of a subset of data which have been used in a previous study by our research team [19]. The previous study found that preoperative NT-proBNP concentrations were independently associated with the risk of AKI after noncardiac surgery and could provide predictive information for AKI after noncardiac surgery. The data of all participants were collected retrospectively from the hospital’s perioperative data warehouse, which is a collaborative programme between Shanghai Lejiu Healthcare Technology Co., Ltd and Southern Medical University Nanfang Hospital. The data warehouse contains demographic characteristics, preoperative medical and medication history, laboratory findings and surgical characteristics for all patients undergoing surgery in the Department of Anesthesiology since 2008. We have considered all adult patients who, from February 2008 through May 2018, underwent major or intermediate noncardiac surgical procedures [20] under regional or general anaesthesia at our hospital. Included patients were required to have serum creatinine, NT-proBNP concentrations, and LVEF levels’ measurements within 30 days before surgery and at least one serum creatinine measurement within 7 days of surgery. For patients who have underwent multiple surgeries during a single hospital stay, only the first surgery was considered. The exclusion criteria for this study were as follows: (1) patients who underwent minor surgical procedure as defined by *the 2014 ESC/ESA guidelines on non-cardiac surgery: Cardiovascular assessment and management *[20]. (2) patients who underwent cardiac, neurological, vascular, urological, ophthalmology, transplant or obstetric procedures; (3) patients with an estimated glomerular filtration rate < 15 ml min-1 1.73 m2; (4) patients with acute kidney injury diagnosed by a clinician before surgery; (5) patients with a history of renal replacement therapy or kidney transplantation [19].

### Variables

Baseline factors potentially associated with postoperative AKI were selected based on literature review. We collected the patients’ preoperative demographic characteristics (sex, age, body mass index), preoperative medical and medication histories (hypertension, congestive heart failure, diabetes, coronary heart disease, ascites, stroke or transient ischaemic attack, the use of renin–angiotensin–aldosterone system inhibitors), laboratory findings (the estimated glomerular filtration rate, serum albumin, proteinuria, haemoglobin), surgical characteristics (type and duration of surgery, type of anaesthesia, emergency surgery or not, major or intermediate surgery [20]) and American Society of Anesthesiologists physical status as the baseline information [19]. The preoperative estimated glomerular filtration rate was estimated preoperatively base on the most recent serum creatinine values using a modified Modification of Diet in Renal Disease equation [21].

Preoperative serum NT-proBNP concentrations were measured in the laboratory department of the hospital using a Roche Elecsys NT-proBNP assay (Roche Diagnostics, Shanghai, China). LVEF level was also measured by a Toshiba Apollo 600. The examinations were completed by certified sonographers. If more than 1 echocardiographic measurement existed, the most recent preoperative measurement was used. Measurements of NT-proBNP concentrations and LVEF levels were ordered based on clinical indications or for preoperative cardiac risk assessment. For patients who had multiple measurements of preoperative NT-proBNP concentrations and LVEF levels, the most recent values were used.

AKI was defined by the Kidney Disease: Improving Global Outcomes (KDIGO) criteria (increase in serum creatinine of ≥ 26.5 μmol l^−1^ within 48 h or ≥ 1.5 times the baseline value within 7 days after surgery) [22].

### Statistical analysis

Continuous variables are presented as medians and interquartile ranges, and categorical variables are presented as counts and percentages. We compared continuous variables with the Mann–Whitney U test and categorical variables with the *χ*^*2*^ or Fisher exact test. Because the distribution of NT-proBNP concentrations was skewed, it was transformed by taking the natural logarithm (ln) [19]. We performed multivariate logistic regression analysis to construct the AKI prediction model with and without NT-proBNP concentrations and LVEF levels.

We calculated the odds ratio (OR) and 95% confidence interval (CI) of all baseline independent variables in both prediction models to determine the predictive significance of preoperative NT-proBNP concentrations and LVEF levels for postoperative acute kidney injury and to assess whether they are independent risk factors. The discrimination ability of the prediction model when preoperative NT-proBNP concentrations and LVEF levels were included was compared by using DeLong’s method to test the change in the area under the receiver operating characteristic curve (AUROC). To evaluate the impact of preoperative NT-proBNP concentrations and LVEF levels on AKI risk prediction, we determined the continuous net reclassification index (NRI) and the integrated discrimination improvement (IDI) index values [23, 24]. Due to the lack of clearly predefined clinical risk thresholds for postoperative AKI, the categorical NRI was not used. CIs are reported for the AUROC and reclassification indices (continuous NRI, IDI, relative IDI). All baseline variables with NT-proBNP concentrations and LVEF levels were analysed by forward stepwise method for multivariate logistic regression analysis. All significant variables were selected to construct the final model for postoperative AKI prediction. The calibration of the model was tested by the Hosmer and Lemeshow goodness of fit (GOF) test.

Statistical analyses were performed using Rstudio Version 1.1.383, MedCalc Version 19.0.7 and SPSS software Version 22. All statistical tests were 2-tailed.

## Results

### Characteristics of the study cohort

**Fig. 1:**
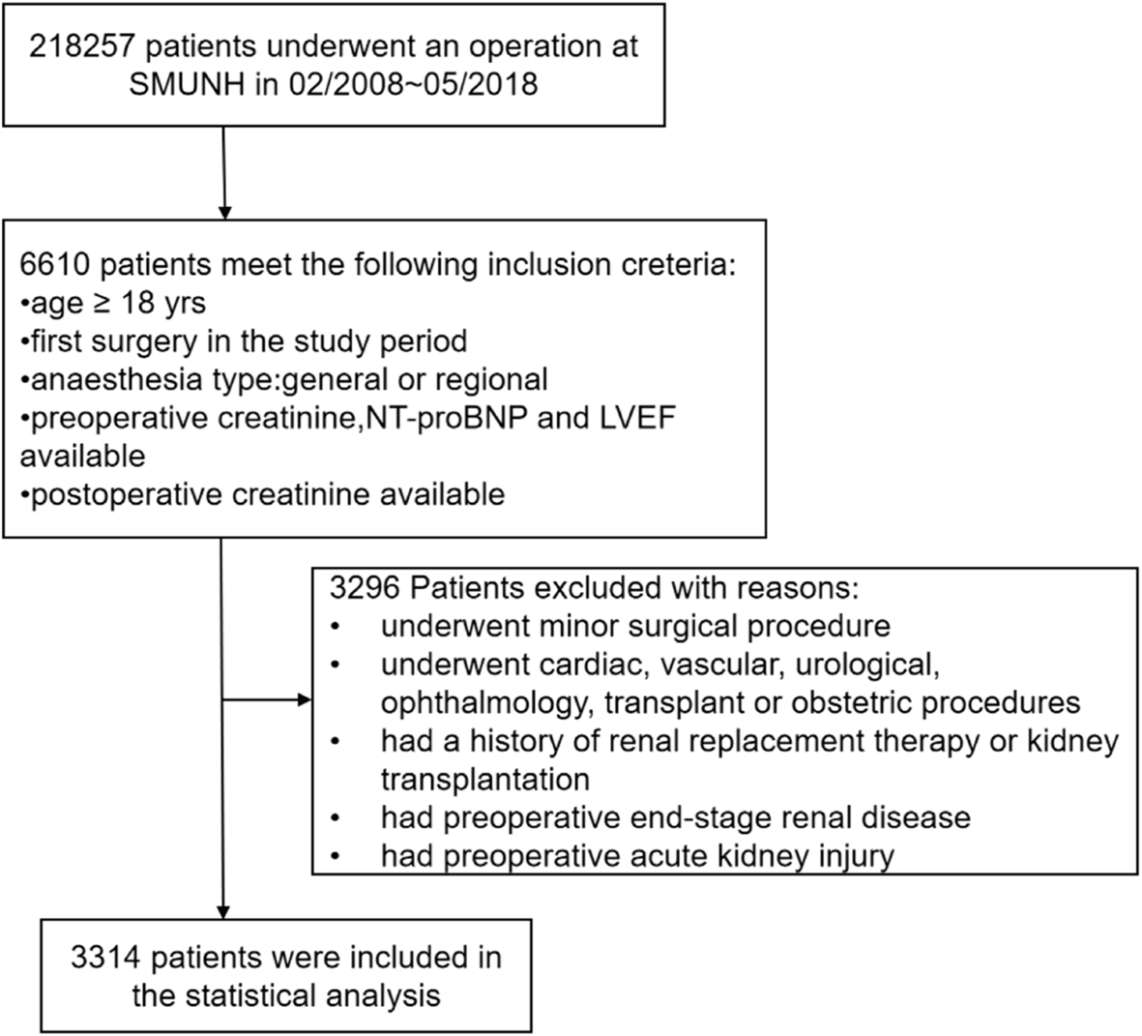
Flow chart of patient selection. Abbreviations: NT-proBNP N-terminal pro-brain natriuretic peptide, LVEF Left ventricular ejection fraction, SMUNH Southern Medical University Nanfang Hospital

Among the 3,314 patients enrolled in this study (the patient selection process is shown in Fig. 1), 6.72% (223 of the 3,314 patients) developed AKI within 1 week after surgery. The baseline characteristics of the patients, both overall and stratified by the presence of postoperative AKI, are provided in Table 1. The median age was 67 years (interquartile range 60 to 73 years), and 52.2% of patients in the cohort were male. Patients who developed AKI were older (70 years, interquartile range 63 to 75 years) and more likely to be male (141 males, 63.2%) and more likely to have more baseline comorbidities. Hypertension, diabetes, stroke, coronary heart disease and congestive heart failure were the most common noncardiac comorbidities. In this study, patients who underwent the minor surgeries were excluded. Among the patients included in the study, the proportion of patients with postoperative AKI who underwent major surgery was higher than that of patients without postoperative AKI.

**Table 1:** Characteristics of the study patients according to the occurrence of postoperative AKI

	Overall	No AKI	AKI	
Variables ^*^	(*n* = 3314)	(*n* = 3091, 93.27%)	(*n* = 223, 6.72%)	*P* value
Age, yrs	67 (60, 73)	66 (60, 73)	70 (63, 75)	< 0.001
Male sex	1729 (52.2)	1588 (51.4)	141 (63.2)	< 0.001
BMI, kg/m^2^	22.86 (20.43, 25.40)	22.86 (20.44, 25.39)	22.50 (20.25, 25.62)	0.617
Medical history				
Hypertension	1161 (35.0)	1037 (33.5)	124 (55.6)	< 0.001
Diabetes	664 (20.0)	595 (19.2)	69 (30.9)	< 0.001
Coronary heart disease	217 (6.5)	175 (5.7)	42 (18.8)	< 0.001
Stroke	171 (5.2)	150 (4.9)	21 (9.4)	0.005
Congestive heart failure	148 (4.5)	102 (3.3)	46 (20.6)	< 0.001
Ascites	25 (0.8)	21 (0.7)	4 (1.8)	0.145
Use of RAAS inhibitors	424 (12.8)	365 (11.8)	59 (26.5)	< 0.001
Preoperative laboratory findings				
eGFR, mL min^−1^ 1.73 m^2^	89.40 (74.53, 105.70)	89.80 (75.50, 105.70)	80.80 (61.55, 106.70)	< 0.001
^ a^Proteinuria (≥ 1 +)	158 (4.8)	125 (4.0)	33 (14.8)	< 0.001
Haemoglobin, g l^−1^	124 .00(110.00, 136.00)	125.00 (110.00, 137.00)	117.00 (101.00, 131.50)	< 0.001
Serum albumin, g l^−1^	37.70 (34.70, 40.60)	37.80 (34.80, 40.60)	37.00 (33.65, 39.50)	0.001
NT-proBNP, pg ml^−1^	76.36 (39.86, 178.47)	72.74 (38.84, 164.60)	163.10 (77.75, 913.50)	< 0.001
LVEF, %	66.00 (62.00, 70.80)	66.00 (62.00, 71.00)	61.00 (50.00, 67.15)	< 0.001
Surgical characteristics				
Emergency surgery	82 (2.5)	66 (2.1)	16 (7.2)	< 0.001
Type of surgery				< 0.001
General	1330 (40.1)	1214 (39.3)	116 (52.0)	
Orthopaedic	1132 (34.2)	1059 (34.3)	73 (32.7)	
Thoracic	786 (23.7)	757 (24.5)	29 (13.0)	
Gynaecological	52 (1.6)	49 (1.6)	3 (1.3)	
Others	14 (0.4)	12 (0.4)	2 (0.9)	
Surgery duration, hrs	2.00 (2.00, 3.00)	2.00 (2.00, 3.00)	2.00 (2.00, 4.00)	0.069
Major surgery	482 (14.5)	438 (14.2)	44 ( 19.7)	0.030
Type of anaesthesia				
General	2820 (85.1)	2626(85.0)	194 (87.0)	0.466
ASA physical status				
I	374 (11.3)	365 (11.8)	9 (4.0)	
II	2397 (72.3)	2273 (73.5)	124 (55.6)	
III	507 (15.3)	429 (13.9)	78 (35.0)	
IV	36 (1.1)	24 (0.8)	12 (5.4)	

*Abbreviations*: *AKI A*cute kidney injury, *ASA *American Society of Anesthesiologists, *BMI *Body mass index, *eGFR E*stimated glomerular filtration rate, *IQR *Interquartile range, *NT-proBNP *N-terminal pro-brain natriuretic peptide, RAAS Renin–angiotensin–aldosterone system

^*^Categorical variables are shown as counts (percentages), and continuous variables are shown as medians (interquartile ranges)

^a^ Defined by a positive (≥ 1 +) urine dipstick test

The median overall preoperative NT-proBNP concentration was 76.36 pg ml^−1^ (interquartile range 39.86 to 178.47 pg ml^−1^), and the preoperative NT-proBNP concentrations were markedly higher in patients who developed AKI after surgery than among those who did not (median 163.10 versus 72.74 pg ml^−1^, *P* < 0.001, Table 1). The median preoperative LVEF level was lower in patients who developed AKI after surgery than among those who did not (median 61.00% versus 66.00%, *P* < 0.001, Table 1).

### Associations between baseline variables and AKI

In the base AKI prediction model containing baseline variables but not NT-proBNP concentrations and LVEF levels, the aORs and 95% CIs of 7 predictors, including sex, ASA physical status, coronary heart disease, congestive heart failure, hypertension, the use of RAAS inhibitors,the duration of surgery, and proteinuria, were greater than 1 (all *P* values < 0.05). The aOR and 95% CI of thoracic surgery were less than 1, and the *P* value was < 0.05. When NT-proBNP concentrations and LVEF levels were added to the base model, the aORs and 95% CIs of above-mentioned 7 predictors were still greater than 1, and all *P* values were < 0.05 (Table 2).

**Table 2:** Multivariable logistic regression models for the prediction of postoperative AKI

Variables	Base model^a^		Base model^†^ + NT-proBNP^*^ and LVEF	
	aOR (95% CI)	*P* value	aOR (95% CI)	*P* value
Age	1.005(0.989, 1.021)	0.574	0.998(0.982, 1.015)	0.813
Male sex	1.815(1.315, 2.505)	0.000	1.470(1.043, 2.071)	0.028
BMI	1.009(0.967, 1.051)	0.689	1.002(0.959, 1.046)	0.939
Medical history				
Hypertension	1.665(1.192, 2.325)	0.003	1.517(1.067, 2.157)	0.020
Diabetes	1.279(0.910, 1.798)	0.157	1.167(0.806, 1.688)	0.414
Coronary heart disease	1.922(1.246, 2.966)	0.003	1.689(1.052, 2.712)	0.030
Stroke	0.914(0.525, 1.592)	0.751	0.787(0.432, 1.436)	0.435
Congestive heart failure	3.221(2.038, 5.091)	0.000	2.401(1.436, 4.014)	0.001
Ascites	2.571(0.760, 8.701)	0.129	2.448(0.713, 8.402)	0.155
Use of RAAS inhibitors	1.589(1.082, 2.332)	0.018	1.603(1.066, 2.410)	0.023
Preoperative laboratory findings				
eGFR	0.997(0.992, 1.003)	0.336	0.998(0.992, 1.004)	0.513
Proteinuria (≥ 1 +)	2.020(1.239, 3.292)	0.005	1.987(1.177, 3.355)	0.010
Haemoglobin	0.994(0.986, 1.002)	0.146	0.997(0.989, 1.006)	0.543
Serum albumin	1.016(0.982, 1.051)	0.365	1.024(0.987, 1.062)	0.199
Surgical characteristics				
Emergency surgery	1.582(0.799, 3.129)	0.188	1.611(0.792, 3.278)	0.188
Type of surgery				
General			Reference	
Orthopaedic	1.028(0.687, 1.538)	0.894	1.021(0.664, 1.568)	0.926
Thoracic	0.608(0.386, 0.957)	0.032	0.592(0.366, 0.957)	0.032
Gynaecological	1.017(0.291, 3.558)	0.979	0.841(0.225, 3.143)	0.796
Others	2.665(0.528, 13.448)	0.235	1.338(0.214, 8.364)	0.756
Surgery duration	1.130(1.016, 1.256)	0.024	1.154(1.032, 1.291)	0.012
Major surgery	1.404(0.940, 2.096)	0.098	1.377(0.901, 2.103)	0.139
Type of anaesthesia				
General	1.440(0.846, 2.449)	0.179	0.648(0.368, 1.139)	0.132
ASA physical status				
I			Reference	
II	1.838(0.912, 3.707)	0.089	1.881(0.902, 3.922)	0.092
III	3.119(1.466, 6.635)	0.003	2.564(1.155, 5.692)	0.021
IV	5.984(2.020, 17.730)	0.001	6.124(1.858, 20.184)	0.003
ln(NT-proBNP)			1.269(1.101, 1.463)	0.001
LVEF			0.911(0.895, 0.928)	0.000

*Abbreviations*: *AKI *Acute kidney injury, *ASA *American Society of Anesthesiologists, *aOR *Adjusted odds ratio, *CI *Confidence interval, *NT-proBNP *N-terminal pro-brain natriuretic peptide, *LVEF *Left ventricular ejection fraction

^*^ Natural log-transformed NT-proBNP

^a^Adjusted for age (years), sex, body mass index, ASA physical status, hypertension, diabetes, coronary heart disease, stroke, congestive heart failure, ascites, the use of renin–angiotensin–aldosterone system inhibitors, emergency surgery, surgery type, surgery duration, anaesthesia type, estimated glomerular filtration rate (continuous), proteinuria, haemoglobin and serum albumin

### Associations between NT-proBNP concentrations, LVEF levels and AKI

When patient demographics, medical history, laboratory findings, surgical characteristics, NT-proBNP concentrations and LVEF levels were included in the logistic model, we found that preoperative NT-proBNP concentrations and LVEF levels were significantly and independently associated with AKI risk. The aOR and 95% CI of preoperative NT-proBNP concentrations were greater than 1 (adjusted OR: 1.269, 95% CI 1.001–1.463, *P* = 0.001, Table 2). The aOR and 95% CI of LVEF levels were less than 1 (adjusted OR: 0.911, 95% CI 0.895–0.928, *P* < 0.001, Table 2).

### Additive value of NT-proBNP concentrations and LVEF levels in risk prediction

The AUROC of the postoperative AKI prediction models with (0.811, 95% CI 0.779–0.843, Table 3) and without preoperative NT-proBNP concentrations and LVEF levels (0.767, 95% CI 0.732–0.802, Table 3) showed statistical significance (*P* < 0.001). The addition of NT-proBNP concentrations and LVEF levels to the base model also improved reclassification by 22.9% (95% CI 10.5–34.4%) for patients who developed postoperative AKI and by 36.3% (95% CI 29.5–43.9%) for those who did not, resulting in a significant overall improvement in net reclassification (NRI 0.591 95% CI 0.437, 0.752, *P* < 0.001). The absolute IDI was 0.100 (95% CI: 0.075, 0.125, *P* < 0.000).

**Table 3:** Performance metrics of postoperative AKI prediction models with and without preoperative NT-proBNP concentrations and LVEF levels

	Base model^a^	Base model^a^ + NT-proBNP^*^ and LVEF
Multivariable logistic regression model ^a^ as the base model		
AUC	0.767 (95% CI: 0.732, 0.802)	0.811 (95% CI: 0.779, 0.843)
ΔAUC	Reference	0.044, *P* < 0.001
Specificity, Sensitivity	0.721, 0.717	0.794, 0.700
NRI for event	Reference	0.229 (95% CI: 0.105, 0.344)
NRI for nonevent	Reference	0.363 (95% CI: 0.295, 0.439)
NRI	Reference	0.591 (95% CI: 0.437, 0.752), *P* < 0.000
IDI	Reference	0.100 (95% CI: 0.075, 0.125), *P* < 0.000

*Abbreviations*: *AKI *Acute kidney injury, *ASA *American Society of Anesthesiologists, *AUC *Area under the curve, *CI *Confidence interval, *IDI *Integral discrimination improvement, *NRI *Net reclassification improvement, *NT-proBNP *N-terminal pro-brain natriuretic peptide, *LVEF *Left ventricular ejection fraction

^*^Natural log-transformed NT-proBNP

^a^Adjusted for age (years), sex, body mass index, ASA physical status, hypertension, diabetes, coronary heart disease, stroke, congestive heart failure, ascites, the use of renin–angiotensin–aldosterone system inhibitors, emergency surgery, surgery type, surgery duration, anaesthesia type, estimated glomerular filtration rate (continuous), proteinuria, haemoglobin and serum albumin

### The final model for the prediction of acute kidney injury after noncardiac surgery

**Fig. 2:**
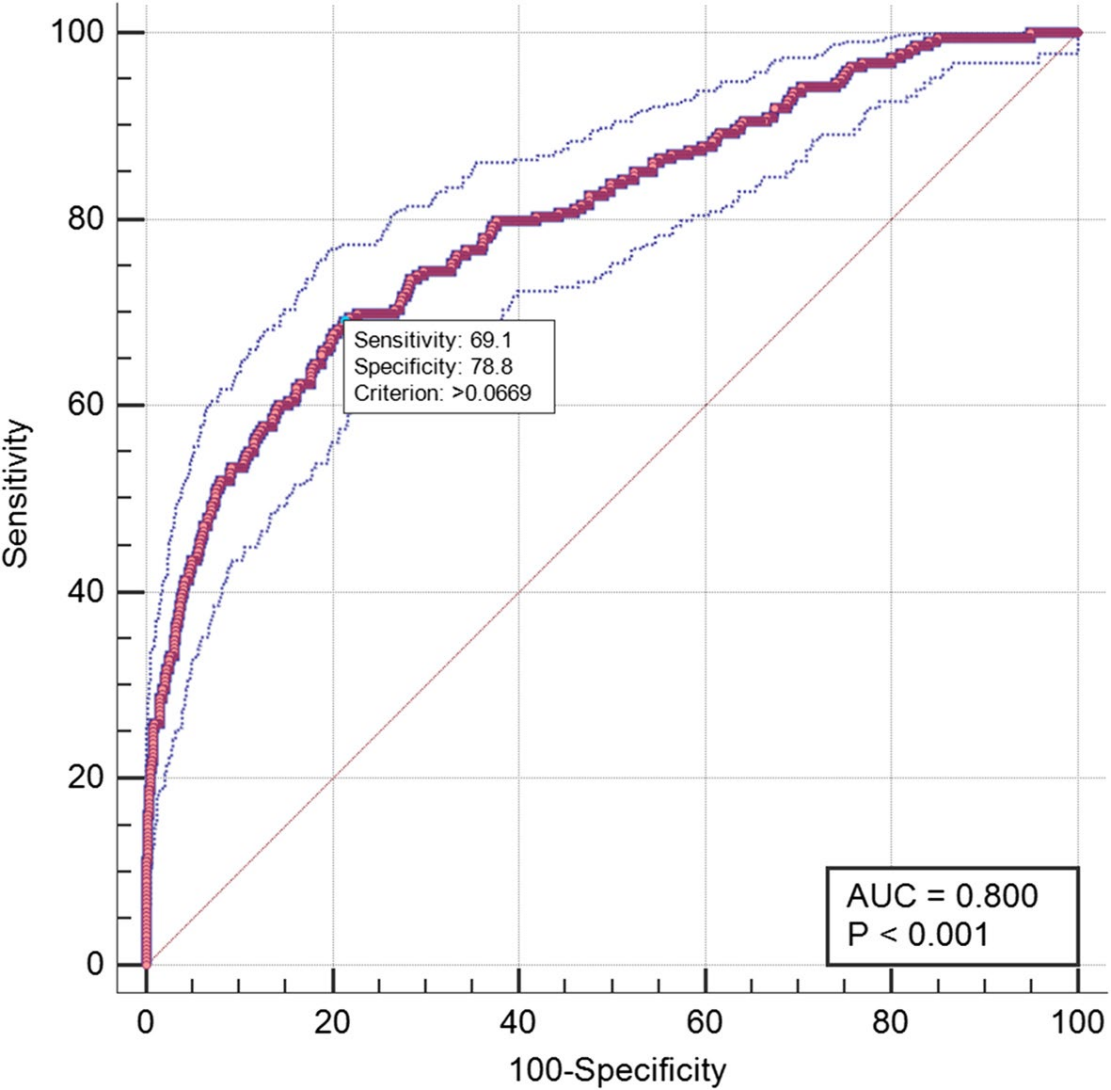
Receiver operating characteristic curve of the final postoperative AKI prediction model

All baseline variables with NT-proBNP concentrations and LVEF levels were analysed by forward stepwise method for multivariate logistic regression analysis. All significant variables were selected for the final model (Table 4), including sex, ASA physical status, coronary heart disease, congestive heart failure, hypertension, the use of RAAS inhibitors,the duration of surgery, proteinuria, NT-proBNP concentrations and LVEF levels. The final postoperative AKI prediction model had a good discriminative ability with AUROC = 0.800 ( 95% CI 0.766–0.833, Fig. 2), and fitted to the dataset by GOF (*χ*^*2*^ = 12.042, *P* = 0.15).

**Table 4:** Performance metrics of the final postoperative AKI prediction model

Variables	The final postoperative AKI prediction model	
	aOR (95% CI)	*P* value
Male sex	1.397(1.017, 1.918)	0.039
Medical history		
Hypertension	1.626(1.163, 2.273)	0.004
Coronary heart disease	1.649(1.045, 2.600)	0.031
Congestive heart failure	2.482(1.497, 4.113)	0.000
Use of RAAS inhibitors	1.494(1.005, 2.220)	0.047
Preoperative laboratory findings		
Proteinuria (≥ 1 +)	2.131(1.297, 3.501)	0.003
Surgical characteristics		
Surgery duration	1.192(1.075, 1.323)	0.001
ASA physical status		
I	Reference	
II	1.807(0.874, 3.737)	0.111
III	2.530(1.155, 5.540)	0.020
IV	6.494(2.039, 20.689)	0.002
ln(NT-proBNP)	1.291(1.137, 1.465)	0.000
LVEF	0.912(0.896, 0.929)	0.000

Variables including sex, ASA physical status, coronary heart disease, congestive heart failure, hypertension, the use of RAAS inhibitors,the duration of surgery, proteinuria, NT-proBNP concentrations and LVEF levels

*Abbreviations*: *AKI *Acute kidney injury, *ASA *American Society of Anesthesiologists, *aOR *Adjusted odds ratio, *CI *Confidence interval, *NT-proBNP *N-terminal pro-brain natriuretic peptide, *LVEF *Left ventricular ejection fraction

## Discussion

NT-proBNP concentrations and LVEF levels are important predictive markers of adverse cardiovascular events after noncardiac surgery [18, 25–28]. Guidelines on perioperative cardiac risk assessment recommend measuring NT-proBNP concentrations and LVEF levels in at-risk patients before noncardiac surgery. NT-proBNP concentrations and LVEF levels are increasingly readily available to anaesthesiologists and surgeons before surgery, especially in patients with more cardiovascular comorbidities. However, there are more current studies on the associations between NT-proBNP concentrations and LVEF levels and AKI after cardiac surgery than before noncardiac surgery. Only a few reports have indicated that postoperative AKI is associated with BNP concentrations or LVEF levels in a single type of noncardiac surgery [17, 18, 29]. Our study shows that in a heterogeneous group of patients undergoing mixed types of noncardiac surgery, preoperative NT-proBNP concentrations and LVEF levels are independently associated with postoperative AKI. This result is also consistent with previous reports that NT-proBNP concentration and LVEF level were associated with the risk of AKI in certain settings [18, 19, 30–35].

For the convenience of discussion, the postoperative AKI prediction models with and without preoperative NT-proBNP concentrations and LVEF levels are defined as the compound model and the base model, respectively. As shown by the statistically significant improvements in the C statistics (AUC), continuous NRI and IDI, we found that adding preoperative NT-proBNP concentrations and LVEF levels to the base model improved AKI risk prediction in our study cohort. In the two preoperative risk prediction models constructed in this study, the C statistics were both > 0.75, which indicated good prediction abilities, and the C statistic of the compound model was significantly higher than that of the base model (*P* < 0.05). In the previous risk prediction model of AKI after noncardiac surgery established by Kheterpal et al. [36] and Bell et al. [3], the C statistics ranged from 0.70 to 0.85. In our study, the C statistic was also within this range, indicating that the predictive ability is comparable to that of the previously studied models.

The mechanism by which NT-proBNP concentration and LVEF level predict AKI is currently unclear. An increase in the NT-proBNP concentration and a decrease in the LVEF level may be caused by a patient's increased venous pressure, diastolic filling pressure, and abnormal ventricular systolic function, and the occurrence of these conditions may in turn antagonize the expansion of circulating blood vessels and act on the renin–angiotensin–aldosterone system, which may lead to impaired kidney function. In this study, we constructed the final model, including sex, ASA physical status, coronary heart disease, congestive heart failure, hypertension, the use of RAAS inhibitors,the duration of surgery, proteinuria, NT-proBNP concentrations and LVEF levels. All these factors may result in different pathophysiological characteristics, which can affect the risk and severity of AKI.

Several limitations of this study should be mentioned here. This study was a retrospective cohort study, and it is uncertain whether preoperative intervention for NT-proBNP concentrations and LVEF levels can reduce the risk of postoperative AKI. The lack of data such as fasting blood glucose, glycosylated haemoglobin, intraoperative fluid rehydration and blood loss and intraoperative urine volume may have affected the evaluation of the risk prediction model. In addition, the inclusion criteria of this study stipulated that the patients must have undergone cardiac colour Doppler ultrasound and NT-proBNP measurement before surgery, which limited the number of patients enrolled to a certain extent and reduced the generalizability of the research model. The study cohort was biased towards a population with more cardiovascular comorbidities than the overall noncardiac surgical population, which probably had a higher AKI risk. These untested patients were likely to be younger, have fewer complications and lower preoperative creatinine levels. Therefore, whether the NT-proBNP- and LVEF-based preoperative risk prediction model is applicable to a relatively healthy population needs to be tested in a prospective trial.

## Conclusions

In this single-centre retrospective study, we found that preoperative NT-proBNP concentrations and LVEF levels were independently associated with the risk of AKI after noncardiac surgery, and they can improve the predictive ability of logistic regression models based on conventional clinical risk factors. Whether preoperative assessment and intervention based on NT-proBNP concentrations and LVEF levels can reduce the incidence of postoperative AKI requires further prospective studies.

## Data Availability

The datasets generated and analysed during the current study are not publicly available due to the protection of the participants' rights to privacy but are available from the corresponding author upon reasonable request.

## Abbreviations

AKI: Acute kidney injury
NT-proBNP: N-terminal pro-brain natriuretic peptide
LVEF: Left ventricular ejection fraction
AUROC: Area under the receiver operating characteristic curve
KDIGO: Kidney Disease: Improving Global Outcomes
OR: Odds ratio
CI: Confidence interval
GOF: Hosmer and Lemeshow goodness of fitnet
IDI: Integral discrimination improvement
NRI: Net reclassification improvement
SMUNH: Southern Medical University Nanfang Hospital
ASA: American Society of Anesthesiologists
BMI: Body mass index
BP: Blood pressure
eGFR: Estimated glomerular filtration rate
IQR: Interquartile range
RAAS: Renin-angiotensin-aldosterone system
aOR: Adjusted odds ratio
CI: Confidence interval
AUC: Area under the curve
